# A screening instrument to identify ulcerative colitis patients with the high possibility of current non-adherence to aminosalicylate medication based on the Health Belief Model: a cross-sectional study

**DOI:** 10.1186/s12876-014-0220-z

**Published:** 2014-12-19

**Authors:** Aki Kawakami, Makoto Tanaka, Masakazu Nishigaki, Naoki Yoshimura, Ryoichi Suzuki, Shin Maeda, Reiko Kunisaki, Noriko Yamamoto-Mitani

**Affiliations:** Inflammatory Bowel Disease Center, Yokohama City University Medical Center, 4-57 Urafune-cho, Minami-ku, Yokohama 232-0024 Japan; Department of Gastroenterology, Yokohama City University Graduate School of Medicine, 3-9 Fukuura, Kanazawa-ku, Yokohama 236-0004 Japan; Department of Advanced Clinical Nursing, Graduate School of Medicine, the University of Tokyo, 7-3-1 Hongo, Bunkyo-ku, Tokyo 113-0033 Japan; Department of Adult Nursing, Graduate school of Medicine the University of Tokyo, 7-3-1 Hongo, Bunkyo-ku, 113-0033 Japan; Department of Gastroenterology, Social Insurance Central General Hospital, 3-22-1 Hyakuninn-cho, Shinjyuku-ku, Tokyo 169-0073 Japan; Kannai Suzuki Clinic, 3-28 Onoue-cho, Naka-ku, Yokohama 231-0028 Japan

**Keywords:** Ulcerative colitis, Medication adherence, Aminosalicylates, Health belief model, Screening

## Abstract

**Background:**

Non-adherence to aminosalicylates is observed among 30% to 45% of patients with ulcerative colitis and increases the risk of relapse. The Health Belief Model is a theoretical model that could offer a broader perspective to improve patients’ self-medication adherence. This study aimed to develop a screening instrument based on the Health Belief Model to screen patients with ulcerative colitis who had a high possibility of current non-adherence to aminosalicylates. The study was also designed to allow examination of factors of non-adherence.

**Methods:**

A multicenter, cross-sectional study was conducted in outpatients diagnosed with ulcerative colitis and prescribed aminosalicylates. Non-adherence was defined as taking less than 80% of the prescribed dose. We hypothesized that there was a significant relationship between current aminosalicylate non-adherence and five components of the HBM: beliefs about taking aminosalicylates, disease characteristics, medication characteristics, abdominal symptoms, and sociodemographic characteristics. A logistic regression model was applied and the coefficients converted to a numeric scores in order to develop a screening instrument which could reliably discriminate non-adherent and adherent subjects.

**Results:**

Non-adherence was observed in 127 (29.6%) of the 429 enrolled subjects. Lower perceptions of belief in taking aminosalicylates, absence of visible bleeding, eight daily tablets or less taken, and no concomitant use of thiopurines were related to non-adherence. We then developed a screening instrument comprising 22 items. When the cut-off point was set at 60, the instrument showed 85.0% sensitivity and 69.2% specificity with an area under the curve of 0.84 (95% confidence interval = 0.79–0.91).

**Conclusions:**

The instrument appeared to be reliable for identifying patients with a high possibility of current non-adherence to aminosalicylates. Further, the instrument may provide useful information for detecting patients with a high possibility of current non-adherence and for assessing factors of non-adherence. On the other hand, we need to evaluate disease activity more strictly and examine whether it is included in the screening instrument in the future.

**Electronic supplementary material:**

The online version of this article (doi:10.1186/s12876-014-0220-z) contains supplementary material, which is available to authorized users.

## Background

Ulcerative colitis (UC) is a chronic, idiopathic, inflammatory bowel disease (IBD) of the large intestine and its disease course is characterized by periods of relapse and remission. Multiple studies have reported the efficacy of aminosalicylates as first-line therapy for inducing remission and preventing UC relapses [1,2]. Non-adherence to aminosalicylates, defined as taking less than 80% of the prescribed dose, was reported by 30% to 45% of patients [3,4] and increases the risk of clinical relapse from 2.3- to 5.5-fold [3,5,6]. A systematic review indicated that a patient’s beliefs about taking medication were consistently associated with non-adherence [4]. Identifying patients with the high possibility of current non-adherence and assessing the individual’s beliefs about taking aminosalicylates seem to be key steps toward improving medication adherence.

In real clinical settings, however, it is often difficult to learn about each patient’s non-adherence or beliefs about taking medication. Many physicians try to screen via interview [7], but patients often prefer not to disclose their actual state to physicians face to face, resulting in non-adherent patients being missed and not receiving further assistance. Thus, it is necessary to developing a screening instrument that allows healthcare providers to identify patients who are likely to be currently non-adherent and assess factors relating to non-adherence.

In examining related factors of non-adherence, a previous review has shown that health behavioral theories enable healthcare providers to understand the relationship between patients’ beliefs and non-adherence [8]. The Health Belief Model (HBM) is one theory that can offer greater insight into motivations to adopt adequate healthcare behavior such as taking medication [9-11]. The HBM identified five domains within one’s belief system as basis for behavior: 1) perceived susceptibility or vulnerability to the disease process; 2) perceived severity of the condition; 3) perceived benefits (belief in efficacy); 4) perceived barriers; and 5) cues to action. The HBM has been widely used as a theoretical framework for interventions or evaluations that attempt to influence healthcare behaviors across a broad range of conditions [12,13]. Previous studies also found that all elements of the HBM were related to medication adherence [12,14,15]. Assessing factors contributing to non-adherence based on the HBM in patients with UC would be an important contribution to the development of interventions to improve medication adherence and prevent relapse.

There is only one instrument focusing on patients’ beliefs around medications. Horne et al. developed the Beliefs about Medicines Questionnaire to assess two subordinate concepts, which are necessity and concerns about taking medications [16]. It has been reported that low adherence has been associated with doubts about personal need for maintenance treatment in IBD and concerns about potential adverse effects [17-19]. Other instruments on medication adherence examine reasons for non-adherence [20-27], or screen patients with high risk of non-adherence [20,27]. These instruments do not typically cover patients’ beliefs, such as the patient’s perspective regarding their medication or their thoughts on forgetting medicine. Clearly, there is a need to develop a screening instrument to assess the high possibility of non-adherence using a comprehensive, theoretical model. This study aimed to develop a screening instrument, using the HBM, for identifying patients with UC who had the high possibility of current non-adherence to aminosalicylates and assessing factors relating to non-adherence.

## Methods

### Study design and patients

Patients diagnosed as having UC and attending one of the outpatient clinics of three hospitals located in urban Japan were consecutively enrolled in this survey. The recruitment period was from May to December 2012. Inclusion criteria were: 1) met the criteria for UC; 2) were over 20 years old; and 3) had been prescribed aminosalicylates. Patients were excluded if they 1) were intolerant to aminosalicylates; 2) had a history of surgery for UC; 3) were unable to complete the questionnaires; 4) had any serious complication; or 5) participated in other clinical studies at the time of study entry.

This study was a cross-sectional survey using a self-administered questionnaire and review of medical records. After the outpatient visit, the physician introduced the patient to one of the researchers. The researcher explained the study protocol to each patient and written informed consent was obtained before the questionnaire was handed to the patient. The study was approved by the Ethics Committees of Yokohama City University Medical Center, University of Tokyo, and Social Insurance Central General Hospital. Patients were informed that answers to the questionnaire were masked to their physicians in order to reduce the possibility that patients with non-adherence might decline participation or answer their adherence more positively than reality.

### Data collection

#### Aminosalicylate non-adherence

**Figure 1:**
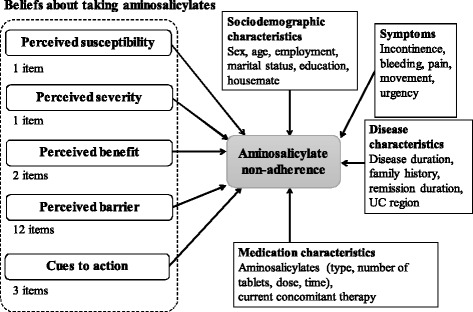
**Initial hypothesized model for current non-adherence in this study.** UC = ulcerative colitis. This is initial hypothesized model for aminosalicylate non-adherence and various factors in our study. We assumed the relationships between aminosalicylates non-adherence and 5 categories: beliefs, sociodemographic characteristics, symptoms, disease characteristics, and medication characteristics.

We hypothesized that there was a significant relationship between current aminosalicylate non-adherence and five components of the HBM: beliefs about taking aminosalicylates, disease characteristics, medication characteristics, abdominal symptoms, and sociodemographic characteristics (Figure 1). The rate of adherence to aminosalicylates during the 7 days prior to study enrollment was investigated using the method employed in previous studies [28]. This method was chosen because we considered it to be the best method to minimize possible recall bias. Subjects answered a question regarding the prescribed numbers of aminosalicylate tablets and missed tablets; the number of tablets was converted to a dose. Prescribed aminosalicylate dose information was also obtained from the subject’s medical record. The aminosalicylate rate of adherence was calculated using the following formula:

$$ \mathrm{Adherence}\kern0.35em \mathrm{rate}=\frac{{\mathrm{D}}_{\mathrm{pres}\kern0.3em \hbox{-} \kern0.3em }{\mathrm{D}}_{\mathrm{miss}}}{{\mathrm{D}}_{\mathrm{pres}}}\times 100 $$

$$ {\mathrm{D}}_{\mathrm{pres}\kern0.35em =}\kern0.35em \mathrm{prescribed}\kern0.35em \mathrm{dose}\kern0.35em \mathrm{o}\mathrm{f}\kern0.35em \mathrm{aminosalicylates}\kern0.35em \mathrm{according}\kern0.35em \mathrm{t}\mathrm{o}\kern0.35em \mathrm{medical}\kern0.35em \mathrm{record} $$

$$ {\mathrm{D}}_{\mathrm{miss}}=\kern0.35em \Big({\mathrm{D}}_{\mathrm{miss}\hbox{-} \mathrm{pt}\kern0.35em +}\kern0.35em {\mathrm{D}}_{\mathrm{pres}\hbox{-} \mathrm{diff}\Big)} $$

$$ {\mathrm{D}}_{\mathrm{miss}\hbox{-} \mathrm{pt}}=\mathrm{doses}\kern0.35em \mathrm{of}\kern0.35em \mathrm{aminosalicylates}\kern0.35em \mathrm{that}\kern0.35em \mathrm{patients}\kern0.35em \mathrm{have}\kern0.35em \mathrm{been}\kern0.35em \mathrm{missed}\kern0.35em \left(\mathrm{quetionnaire}\right) $$

$$ {D_{\mathrm{pres}}}_{\hbox{-} \mathrm{diff}}=\left|{\mathrm{D}}_{\mathrm{pres}},-,\mathrm{prescribed}\kern0.35em \mathrm{dose}\kern0.35em \mathrm{o}\mathrm{f}\kern0.35em \mathrm{aminosalicylates}\kern0.35em \mathrm{according}\kern0.35em \mathrm{t}\mathrm{o}\kern0.35em \mathrm{patiens}\kern0.35em \left(\mathrm{questionaire}\right)\right| $$

Aminosalicylate non-adherence was defined as taking less than 80% of the prescribed doses and adherence as consumption of 80% or more [3,5]. The validity of the 7-day self-report was confirmed using the pill count method over 2 months in a group of 32 subjects, separate from the group of 429 subjects, who were diagnosed with UC, took aminosalicylates, and were enrolled using the same inclusion and exclusion criteria in this study. Most subjects were within the accepted range of agreement: all except 1 were within the 95% confidence interval (CI) according to the Bland-Altman method [29].

#### Beliefs about taking aminosalicylates

An extensive literature review and interviews with research experts in IBD (five physicians, three clinical nurses, and two research nurses) were conducted resulting in the inclusion of 19 items regarding beliefs about taking aminosalicylates. These items were examined by six patients and three IBD specialists for face validity and all concluded that the items were relevant and representative of possible beliefs about taking aminosalicylates. The 19 items were then assigned to each of the five domains of the beliefs about taking aminosalicylates in the HBM: 1 item for “perceived susceptibility”, 1 item for “perceived severity”, 2 items for “perceived benefit”, 12 items for “perceived barrier”, and 3 items for “cues to action” (Figure 1). For items related to “perceived barriers”, we used the items of “difficulties in taking aminosalicylates” that we developed in a previous study [30]. The items were measured with a 5-point Likert-scale, with a higher score indicating a lower perception of susceptibility, severity, or benefit, fewer cues to action, and a higher perception of barrier.

#### Disease, medication, and sociodemographic characteristics and abdominal symptoms

The following information was collected from medical records or asked in the questionnaire: disease characteristics (duration of UC, family history, duration of current remission, disease region by recent colonoscopy); medication characteristics (type of aminosalicylate, times of day at which the drugs were taken, daily number of tablets taken, daily prescribed dose, current concomitant therapy); and abdominal symptoms (stool consistency, bowel movements, urgency, pain, visible bleeding). Quantitative variables were categorized based on previous studies [4], or using their median. Subjects were also asked their sociodemographic characteristics (gender, age, employment, marital status, educational level, and living situation) in the questionnaire.

### Statistical analysis

Descriptive statistics were shown as mean and standard deviation (SD) for continuous variables and as n (%) for categorical variables. After confirming that Cronbach’s alphas of “perceived benefits” and “cues to action” were above 0.7, responses in items were summed to yield the total scores. We conducted a univariate logistic regression analysis to explore factors related to aminosalicylate non-adherence. The variables with possible association with non-adherence (p < 0.2) were entered into the initial multiple logistic regression model. When Spearman’s rho was more than 0.7 among the independent variables, the variable of the smaller p-value was left in the model. A stepwise multiple logistic regression analysis was then conducted. The criterion to remain in the multivariate logistic regression model was set at p < 0.2. The type of hospital was kept in the model as a control variable.

Variables that stayed in the final logistic regression model with p < 0.05 were used in the screening instrument for non-adherence. Calibration of the final model was assessed by the Hosmer-Lemeshow χ^2^ test. Based on standardized coefficients calculated in the logistic regression model, we developed a weighted score as the screening instrument based on Sullivan’s method [31]. Briefly, each coefficient was divided by the smallest coefficient and each quotient rounded to the nearest whole number. Each subject’s score was then calculated by summing up the points of all variables. The sum of these points was used as the screening instrument for non-adherence.

Discriminability of the screening instrument was assessed by drawing a receiver-operating characteristic (ROC) curve and calculating the area under the curve (AUC). The cut-off point for detecting patients with current non-adherence in the screening instrument was determined as a target for the point at the intersection of the inverse cumulative frequency distribution curve and the cumulative frequency distribution curve. The screening instrument was also assigned to differentiate risk classes by quartile, and the prevalence of current non-adherence was compared using the Cochran-Armitage test for trend. All statistical tests were two-tailed, and statistical significance was defined as p < 0.05. All analyses were performed with SAS version 9.3 for Windows (SAS Institute Inc., Cary, NC, USA).

## Results

### Subjects

Four hundred and fifty-five patients met the inclusion criteria. Twenty-six patients were excluded for the following reasons: 8 patients declined entry and 18 were missing more than 20% of items in the questionnaire. Thus, 429 subjects were included in the final analyses. The valid response rate was 95.9% (Table 1). A total of 127 (29.6%) subjects were classified as non-adherent. The mean adherence rate in all subjects was 85.9% with a SD of 17.3. The mean age was 39.9 years, and 56.6% were male. The mean prescribed aminosalicylate dose was 3.5 grams per day. One hundred and sixteen subjects (27.0%) had visible bleeding. Cronbach’s alphas of “cue to action” and “perceived benefits” in beliefs about taking aminosalicylates were 0.72 and 0.71, respectively; therefore responses to those items were summed to yield a total score for each of these domains.

**Table 1:** **Subject characteristics and univariate logistic regression analysis of factors associated with non-adherence (N = 429)**

		**n (%) or Mean ± SD** ^**1**^		**OR** ^**2**^	**95% CI** ^**3**^	**p value**
**Beliefs about taking aminosalicylates**						
Susceptibility [0-4]		1.5 ± 0.9		1.61	1.28–2.02	<0.01
Severity [0-4]		0.7 ± 1.0		2.22	1.78–2.75	<0.01
Benefits [0-8]		2.5 ± 1.7		1.32	1.17–1.49	<0.01
Barriers [0-48]		21.3 ± 9.9		1.11	1.07–1.13	<0.01
Cues to action [0-12]		4.6 ± 2.7		1.20	1.10–1.30	<0.01
**Disease characteristics**						
Duration of UC: Less than 5 years [more than 5 years]		143	(33.4)	1.20	0.76–1.86	0.44
Family history: Absence [presence]		409	(95.3)	0.98	0.37–2.61	0.96
The duration of current remission^a^: More than 3 months [less than 3 months]		330	(77.3)	1.57	0.92–2.66	0.09
Disease region by recent colonoscopy^b^: Rectum [total, left-side]		113	(26.4)	1.54	0.98–2.43	0.06
**Medication characteristics**						
Aminosalicylates						
	Type of aminosalicylates^c^: Mesalazine [salazosulfapyridine]	130	(32.8)	1.02	0.64–1.61	0.94
	Times of day at which the drugs were taken:	278	(64.8)	1.14	0.74–1.77	0.55
	2 times or less [3 times or more]					
	Number of tablets/day: 8 tablets or less [9 tablets or more]	216	(50.3)	1.79	1.18–2.73	<0.01
	Prescribed dose (g/day)	3.5 ± 0.9		0.75	0.59–0.95	0.02
Current concomitant therapy						
	Topical aminosalicylates: Absence [presence]	301	(70.2)	1.11	0.70–1.75	0.66
	Oral corticosteroids: Absence [presence]	404	(94.2)	2.30	0.77–6.84	0.13
	Topical corticosteroids: Absence [presence]	395	(92.1)	1.68	0.71–3.97	0.23
	Thiopurines: Absence [presence]	369	(86.0)	2.32	1.14–4.39	0.02
	Calcineurin inhibitors: Absence [presence]	410	(95.6)	1.19	0.42–3.37	0.78
	Leukocyte apheresis: Absence [presence]	421	(98.1)	1.27	0.25–6.36	0.77
	Biologics: Absence [presence]	403	(93.9)	2.41	0.82–7.16	0.11
**Abdominal symptoms**						
	Stool consistency: Formed [liquid]	375	(87.4)	0.99	0.53–187	0.99
	Bowel movements/day: 3 times or less [4 times or more]	178	(41.5)	1.22	0.80–1.85	0.36
	Urgency: Absence [presence]	239	(55.7)	1.21	0.80–1.85	0.37
	Pain: Absence [presence]	288	(67.1)	0.89	0.58–1.38	0.61
	Visible bleeding: Absence [presence]	313	(73.0)	1.65	1.003–2.71	0.04
**Sociodemographic characteristics**						
Gender: Male [female]		243	(56.6)	0.87	0.58–1.33	0.53
Age		39.9 ± 12.5		0.99	0.97–1.01	0.27
Employment: Full time job, family-operated business [part time job, student, housewife, unemployed]		243	(56.6)	1.32	0.87–2.02	0.19
Marital status: Married [unmarried, divorced, widowed]		251	(41.5)	0.92	0.60–1.41	0.71
Educational level: Junior high school, high school, vocational school, junior college [college or higher]		207	(48.2)	0.93	0.61–1.40	0.71
Living situation: Living alone [living with someone]		68	(15.9)	1.14	0.65–1.98	0.65
Hospital						
	Hospital B [A]	239	(55.7)	1.10	0.65–1.85	0.72
	Hospital C [A]	87	(20.3)	1.72	0.93–3.19	0.10

Information in [brackets] are reference categories.

^a^n = 427, ^b^n = 428, ^c^n = 397 (32 subjects were prescribed two kinds of aminosalicylates and not included in this analysis).

^1^SD: standard deviation, ^2^OR: odds ratio, ^3^CI: confidence interval.

### Related factors for current non-adherence

Based on the results of univariate logistic regression analyses, the following factors were included in the multivariate logistic regression analyses: all domains in the beliefs about taking aminosalicylates; the length of current remission; disease region by recent colonoscopy; daily number of tablets taken; visible bleeding; following current concomitant therapy: oral corticosteroids, thiopurines, and biologics; employment status; (Table 1). Because there was a significant association between daily number of tablets taken and daily prescribed dose (*r*_*s*_ = 0.74, p < 0.01), we used daily number of tablets taken rather than daily prescribed dose.

All domains in the beliefs about taking aminosalicylates remained in the final logistic regression model when other factors were adjusted (Table 2). Other factors remaining in the model were absence of visible bleeding, 8 daily tablets or less taken, and no concomitant use of thiopurines.

**Table 2:** **Multivariate model for non-adherence and risk score (N = 426)**

**Variables**	**Coefficients**	**Odds ratio (95% confidence interval)**	**p value**	**Risk score weights** ^**a**^
Beliefs about taking aminosalicylates				
Susceptibility	0.58	1.78 (1.32–2.42)	<0.01	6
Severity	0.81	2.35 (1.82–3.05)	<0.01	9
Benefits	0.12	1.13 (0.96–1.33)	0.15	1
Barrier	0.09	1.10 (1.06–1.13)	<0.01	1
Cues to action	0.13	1.14 (1.06–1.13)	0.02	1
Visible bleeding				
Absence[presence]	0.90	2.54 (1.33–4.86)	<0.01	10
Current concomitant therapy				
Thiopurines				
Absence [presence]	0.74	2.10 (0.88–5.05)	<0.01	8
Number of tablets/day				
8 tablets or less [9 tablets or more]	0.60	1.92 (1.12–3.30)	0.02	7
Hospital				
Hospital B [A]		0.59 (0.29–1.19)	0.14	
Hospital C [A]		1.81 (0.82–4.00)	0.14	

Information in [brackets] are reference categories. Hosmer-Lemeshow statistics = 9.36 (degree of freedom = 8, p = 0.31).

^a^The method described by Sullivan et al. [31] was used to calculate the risk score weight: Step 1: Divide each regression coefficient by the smallest coefficient in the final logistic regression model (in our model, this is barrier). Step 2: Round this quotient to the nearest whole number. For example, to calculate the score weight of susceptibility, its coefficient of 0.58 was divided by the 0.09, which was the less perceived barrier, resulting in a quotient of 6.44. Rounding this quotient to its nearest integer resulted in 6 for the score weight of this variable. Each subject’s overall screening instrument was then calculated by summing the points of all variables.

### Development of the screening instrument for current non-adherence based on HBM

**Figure 2:**
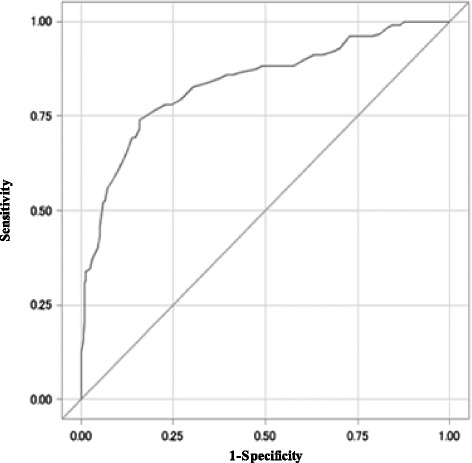
**Receiver operating characteristic curve for the screening instrument for non-adherence.** This is the Receiver Operating Characteristic curve we applied to assess current non-adherence. The area under the curve was 0.84 (95% confidence interval = 0.79 to 0.91). Sensitivity was 85.0% and specificity was 69.2% when a cut-off value of 60 was applied. N = 426.

**Figure 3:**
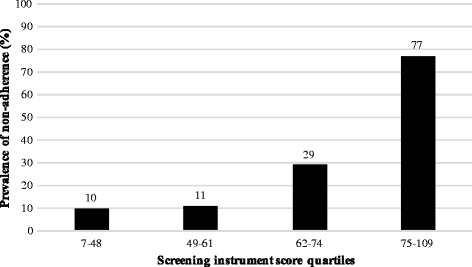
**Observed non-adherence rates by score strata.** The current non-adherence rate increased significantly by risk score strata (p < 0.01 by the Cochran-Armitage test for trend). N = 127.

The risk score weights based on each factor’s coefficient in the final multivariate logistic regression model are shown in Table 2. Each coefficient was divided by the smallest coefficient, which was “perceived barrier”, and each quotient was rounded to the nearest whole number. The screening instrument consists of 22 variables and the range of the overall score was 0 to 153, with a higher score indicating a higher possibility of current non-adherence. The mean score and SD were 61.5 and 18.9 (range: 7–109). The ROC curve of the total score is shown in Figure 2 (AUC = 0.84, 95% CI = 0.79–0.91). The instrument showed a sensitivity of 85.0 and specificity of 69.2 when a cut-off point of 60 was used. When subjects were grouped into fours by quartile score, the current non-adherence rate increased significantly with score strata (p < 0.01) (Figure 3). The final screening instrument is shown in the Additional file 1.

## Discussion

We developed a screening instrument for current aminosalicylate non-adherence and examined factors of non-adherence among patients with UC using the HBM. By using this screening instrument, healthcare providers might be able to identify patients who need support preferentially and simultaneously assess their beliefs around taking aminosalicylates. An additional benefit of this instrument is that patients can complete it by themselves. Our results show that this screening instrument may be useful and contribute to enhancing aminosalicylate adherence in clinical practice.

In this study, the AUC of this non-adherence screening instrument was 0.84 (95% CI = 0.79–0.91), and the non-adherence rate steadily increased with advancing score of the instrument using the quartile groups. These results imply that the instrument has the ability to distinguish between current non-adherent and adherent patients. Further studies using this instrument would confirm its practical validity regarding whether or not it is in fact useful in identifying high risk populations.

All domains in the beliefs about taking aminosalicylates as well as other clinically important factors were identified as related to current non-adherence, underlining the importance of examining these factors for detecting non-adherence. Relationships between patients’ perception and health behaviors have also been reported in previous studies. For example, Janz and Becker found that awareness of susceptibility was one of the predictors (albeit the weakest) of behaviors [11]. Other studies reported that having a perception of susceptibility had a significant effect on appropriate self-care behaviors including medication adherence among patients with chronic illness [13,14], in line with our findings. Higher perceived severity was also strongly associated with adequate self-care among patients with chronic illness [11,12]. Rosenstock noted that the combination of perceived susceptibility and severity has been labeled ‘perceived threat’, with the threat providing the energy or force to act [32]. We should therefore assess patients’ susceptibility and severity, and provide support for the patient to have an appropriate perception of both domains.

On the other hand, our results showed that perceived barrier is a cause of non-adherence. This element proved to be the most powerful of the HBM elements in various studies [15]. Rosenstock also found that the likelihood of action is increased if the benefit of the behavior outweighs the barriers [32]. We should therefore help patients to minimize their perceived barriers to taking medication. For example, memory strategies such as reminders may be useful in preventing a diminished sense of priority for medication. Goal setting techniques may also be effective in reducing barriers in a step-by-step manner [33].

Fewer “cues to action”, (i.e., communication with family or healthcare providers about medication and reminders from them) were another cause of non-adherence. A systematic review showed that patient-physician discordance and lack of trust in one’s physician were strongly associated with non-adherence [34]. Moreover, a previous study reported that chronically ill patients who received support or reminders from their families had better outcomes than patients who did not [35]. Receiving reminders or encouragement from healthcare providers and family members may work as important “cues to action” to improve adherence.

We also found that non-adherence increases when subjects had absence of visible bleeding, fewer than eight daily tablets, and no concomitant use of thiopurines. These results were concordant with some previous studies [4,18]. These conditions taken together suggest a rather stable disease course and therefore the patient may forget to take their medications. This may mean that patients with stable conditions should also be monitored carefully for possible non-adherence.

There are some limitations to this study. The first limitation is selection bias: the study was conducted at IBD specialist clinics in urban areas and subjects may have been well-educated regarding their disease and treatment. For example, the non-adherence rate in this study was about 30%, which is slightly less than in a previous review [4]. We should evaluate the validity of this screening instrument under different circumstances and among different groups of patients to confirm its generalizability. Second, aminosalicylate adherence was assessed using a self-report questionnaire and medical records, and the result may be influenced by recall bias that is inherent in any retrospective study. As there is no gold standard method to measure medication adherence, it is often recommended to assess adherence in multiple ways and to evaluate agreement between them. We conducted the present study after confirming subject-reported adherence using the pill count method. A previous study also reported that the self-reporting method had moderate to high agreement with other methods [36]. Third, we used abdominal symptoms as a surrogate marker for patients’ disease activity in this study. Disease activity is one of the most important factors to assess patients’ non-adherence. Although visible bleeding was adopted as one of the items of our tool to assess non-adherence, it might be better if we could use some validated disease activity index. We need to evaluate disease activity more strictly and examine whether it is included in the screening instrument. Additionally, we set a cut-off point to identify as many non-adherent subjects as possible. Therefore, the false-negative rate was about 14%. However, about 30% of subjects in the good adherence group were identified as having the high possibility of non-adherence (false-positive). In the future, we should consider a different cut-off point depending on the purpose of the instrument. Finally, we developed the instrument to identify patients with a high possibility of current non-adherence rather than predicting the risk of future non-adherence. Further prospective research would be needed to evaluate its ability to predict future non-adherence.

## Conclusions

We have developed a screening instrument for identifying patients with UC who had a high possibility of current aminosalicylate non-adherence and for assessing factors influencing non-adherence, using the framework of the HBM. All dimensions in the HBM were identified as related to current non-adherence. Based on our screening instrument, which had acceptable performance, subjects who were currently non-adherent were identified with high sensitivity. The instrument will provide useful information for detecting subjects with the high possibility of current non-adherence and for assessing them in order to improve their adherence.

## Additional file

Additional file 1
**The screening instrument to identify UC patients with a high possibility of current non-adherence to aminosalicylate medication.** Description of data: This is the screening instrument to identify UC patients with a high possibility of current non-adherence to aminosalicylate medication based on the Health Belief Model. The range of this model is from 0 to 153. A patient whose scores more than 60 is believed to have a high possibility of current non-adherence.

## Abbreviations

UC: Ulcerative colitis
IBD: Inflammatory bowel disease
HBM: Health belief model
CI: Confidence interval
SD: Standard deviation
ROC: Receiver-operating characteristic
AUC: Area under the curve, OR: Odds ratio

## References

[1] Dignass A, Lindsay JO, Sturm A, Windsor A, Colombel JF, Allez M, D'Haens G, D'Hoore A, Mantzaris G, Novacek G, Oresland T, Reinisch W, Sans M, Stange E, Vermeire S, Travis S, Van Assche G (2012). Second European evidence-based consensus on the diagnosis and management of ulcerative colitis part 2: current management. J Crohns Colitis.

[2] Ford AC, Achkar J-P, Khan KJ, Kane SV, Talley NJ, Marshall JK, Moayyedi P (2011). Efficacy of 5-aminosalicylates in ulcerative colitis: systematic review and meta-analysis. Am J Gastroenterol.

[3] Kawakami A, Tanaka M, Nishigaki M, Naganuma M, Iwao Y, Hibi T, Sanada H, Yamamoto-Mitani N, Kazuma K (2013). Relationship between non-adherence to aminosalicylate medication and the risk of clinical relapse among Japanese patients with ulcerative colitis in clinical remission: a prospective cohort study. J Gastroenterol.

[4] Jackson CA, Clatworthy J, Robinson A, Horne R (2010). Factors associated with non-adherence to oral medication for inflammatory bowel disease: a systematic review. Am J Gastroenterol.

[5] Kane S, Huo D, Aikens J, Hanauer S (2003). Medication nonadherence and the outcomes of patients with quiescent ulcerative colitis. Am J Med.

[6] Khan N, Abbas AM, Bazzano LA, Koleva YN, Krousel-Wood M (2012). Long-term oral mesalazine adherence and the risk of disease flare in ulcerative colitis: nationwide 10-year retrospective cohort from the veterans affairs healthcare system. Aliment Pharmacol Ther.

[7] Trindade AJ, Morisky DE, Ehrlich AC, Tinsley A, Ullman TA (2011). Current practice and perception of screening for medication adherence in inflammatory bowel disease. J Clin Gastroenterol.

[8] Haynes BR, Ackloo E, Sahota N, McDonald PH, Yao X: **Interventions for enhancing medication adherence.** *Cochrane Database Sys Rev* 2010:1-159 10.1002/14651858.CD000011.pub3 18425859

[9] Rosenstock IM (1974). Historical origins of the health belief model. Health Educ Monogr.

[10] Becker MH, Maiman LA (1975). Sociobehavioral determinants of compliance with health and medical care recommendations. Med Care.

[11] Janz NK, Becker MH (1984). The health belief model: A decade later. Health Educ Q.

[12] DiMatteo MR, Haskard KB, Williams SL (2007). Health beliefs, disease severity, and patient adherence: a meta-analysis. Med Care.

[13] Harvey JN, Lawson VL (2009). The importance of health belief models in determing self-care behaviour in diabetes. Diabet Med.

[14] Gao X, Nau DP, Rosenbluth SA, Scott V, Woodward C (2000). The relationship of disease severity, health beliefs and medication adherence among HIV patients. AIDS Care.

[15] Carpenter CJ (2010). A meta-analysis of the effectiveness of health belief model variables in predicting behavior. Health Commun.

[16] Horne R, Weinman J, Hankins M (1999). The Beliefs about Medicines Questionnaire (BMQ); the development and evaluation of a new methos for assessing the cognitive representation of medication. Psychol Health.

[17] Horne R, Parham R, Driscoll R, Robinson A (2009). Patients' attitudes to medicines and adherence to maintenance treatment in inflammatory bowel disease. Inflamm Bowel Dis.

[18] Selinger CP, Eaden J, Jones DB, Katelaris P, Chapman G, McDonald C, Smith P, Lal S, Leong RW, McLaughlin JAR (2013). Modifiable factors associated with nonadherence to maintenance medication for inflammatory bowel disease. Inflamm Bowel Dis.

[19] Horne R, Chapman SC, Parham R, Freemantle N, Forbes AVC (2013). Understanding patients' adherence-related beliefs about medicines prescribed for long-term conditions: a meta-analytic review of the Necessity-Concerns Framework. PLoS One.

[20] Trindade AJ, Ehrlich A, Kornbluth A, Ullman TA (2011). Are your patients taking their medicine? Validation of a new adherence scale in patients with inflammatory bowel disease and comparison with physician perception of adherence. Inflamm Bowel Dis.

[21] Brooks CM, Richards JM, Kohler CL, Soong SJ, Martin B, Windsor RA, Bailey WC (1994). Assessing adherence to asthma medication and inhaler regimens: a psychometric analysis of adult self-report scales. Med Care.

[22] Horne R, Weinman J (2002). Self-regulation and self-management in asthma: exploring the role of illness perceptions and tratment beliefs in explaining non-adherence to preventer medication. Psychol Health.

[23] Thompson K, Kulkarni J, Sergejew AA (2000). Reliability and validity of a new medication adherence rating scale (MARS) for the psychoses. Schizophr Res.

[24] Horne R, Weinman J (1999). Patients' beliefs about prescribed medicines and their role in adherence to treatment in chronic physical illness. J Psychosom Res.

[25] Knobel H, Alonso J, Casado JL, Collazos J, Gonzalez J, Ruiz I, Kindelan JM, Carmona A, Juega J, Ocampo A, Group GS (2002). Validation of a simplified medication adherence questionnaire in a large cohort of HIV-infected patients: the GEEMA Study. AIDS.

[26] Morisky DE, Ang A, Krousel-Wood M, Ward HJ (2008). Predictive validity of a medication adherence measure in an outpatient setting. J Clin Hypertens.

[27] Kane S, Becker B, Harmsen WS, Kurian A, Morisky DE, Zinsmeister AR (2012). Use of a screening tool to determine nonadherent behavior in inflammatory bowel disease. Am JGastroenterol.

[28] Simoni JM, Kurth AE, Pearson CR, Pantalone DW, Merrill JO, Frick PA (2006). Self-report measures of antiretroviral therapy adherence: A review with recommendations for HIV research and clinical management. AIDS Behav.

[29] Bland JM, Altman DG (1999). Measuring agreement in method comparison studies. Stat Methods Med Res.

[30] Kawakami A, Tanaka M, Ochiai R, Naganuma M, Iwao Y, Hibi T, Kazuma K (2012). Difficulties in taking aminosalicylates for patients with ulcerative colitis. Gastroenterol Nurs.

[31] Sullivan LM, Massaro JM, D'Agostino RB (2004). Presentation of multivariate data for clinical use: The Framingham Study risk score functions. Stat Med.

[32] Rosenstock IM, Strecher VJ, Becker MH (1988). Social learning theory and the Health Belief Model. Health Educ Q.

[33] Funnell MM, Brown TL, Childs BP, Haas LB, Hosey GM, Jensen B, Maryniuk M, Peyrot M, Piette JD, Reader D, Siminerio LM, Weinger K, Weiss MA (2011). National Standards for diabetes self-management education. Diabetes Care.

[34] Nguyen GC, LaVeist TA, Harris ML, Datta LW, Bayless TM, Brant SR (2009). Patient trust-in-physician and race are predictors of adherence to medical management in inflammatory bowel disease. Inflamm Bowel Dis.

[35] Wood DA, Kotseva K, Connolly S, Jennings C, Mead A, Jones J, Holden A, De Bacquer D, Collier T, De Backer G, Faergeman O, Group ES (2008). Nurse-coordinated multidisciplinary, family-based cardiovascular disease prevention programme (EUROACTION) for patients with coronary heart disease and asymptomatic individuals at high risk of cardiovascular disease: a paired, cluster-randomised controlled trial. Lancet.

[36] Garber MC, Nau DP, Erickson SR, Aikens JE, Lawrence JB (2004). The concordance of self-report with other measures of medication adherence: a summary of the literature. Med Care.

