# Supplementary material for: A screening instrument to identify ulcerative colitis patients with the high possibility of current non-adherence to aminosalicylate medication based on the Health Belief Model: a cross-sectional study
Source: BMC Gastroenterol. 2014 Dec 19;14:220. doi: 10.1186/s12876-014-0220-z (PMC4279902; doi:10.1186/s12876-014-0220-z)
Supplement: Additional file 1 — The screening instrument to identify UC patients with a high possibility of current non-adherence to aminosalicylate medication. Description of data: This is the screening instrument to identify UC patients with a high possibility of current non-adherence to aminosalicylate medication based on the Health Belief Model. The range of this model is from 0 to 153. A patient whose scores more than 60 is believed to have a high possibility of current non-adherence. [file 12876_2014_220_MOESM1_ESM.pdf]

Additional file 1

The screening instrument to identify UC patients with a high possibility of current non-adherence to aminosalicylate medication

	Never	Rarely	Unsure	Somewhat	Definitely
Susceptibility					
I think it would be easy to flare up with my current adherence.	24	18	12	6	0
Severity					
I think that relapse would have an enormous impact on my daily life including work, school, or housework.	36	27	18	9	0
Benefits					
I think I will reduce the risk of relapse if I take it as instructed.	4	3	2	1	0
I think I will reduce the possibility to develop colon cancer if I take it as instructed.	4	3	2	1	0
Barrier					
I miss the scheduled time to take it when I am busy at work, school, or with housework.	0	1	2	3	4
I forget to carry it around when I go out.	0	1	2	3	4
I miss the scheduled time to take it when I eat meals at irregular hours.	0	1	2	3	4
I feel less need to have to take it.	0	1	2	3	4
I hesitate to take it in front of others.	0	1	2	3	4
I feel anxious that it may cause unpleasant side effects.	4	3	2	1	0
I feel anxious about long term-side effects.	4	3	2	1	0
I feel anxious about unpleasant side effects when my condition becomes worse.	4	3	2	1	0
It is difficult to take so many pills.	4	3	2	1	0
It is difficult to swallow such large pills.	4	3	2	1	0
It is difficult to take it so many times.	4	3	2	1	0
It is difficult to take it for a long time.	4	3	2	1	0
Cues to action					
I can talk to healthcare providers about my anxiety or worries about it.	4	3	2	1	0
I am encouraged by healthcare providers or my family to take it as instructed.	4	3	2	1	0
Healthcare providers or my family check whether I took it as instructed.	4	3	2	1	0
Visible bleeding	0. Presence		10. Absence		
Current thiopurines	0. Presence		8. Absence		
Daily number of aminosalicylate tablets	0. 9 or more		7. 8 or less		

The range of this model is from 0 to 153. A patient whose scores more than 60 is believed to have a high possibility of current non-adherence.
